# A 12-week aerobic exercise intervention results in improved metabolic function and lower adipose tissue and ectopic fat in high-fat diet fed rats

**DOI:** 10.1042/BSR20201707

**Published:** 2021-01-29

**Authors:** Venkatesh Gopalan, Jadegoud Yaligar, Navin Michael, Kavita Kaur, Rengaraj Anantharaj, Sanjay Kumar Verma, Suresh Anand Sadananthan, Giang Thi Thu Le, Jorming Goh, S. Sendhil Velan

**Affiliations:** 1 Laboratory of Molecular Imaging, Singapore Bioimaging Consortium, Agency for Science Technology and Research (A*STAR), Singapore; 2 Singapore Institute for Clinical Sciences, Agency for Science Technology and Research (A*STAR), Singapore; 3 Healthy Longevity Translational Research Program, Yong Loo Lin School of Medicine, National University of Singapore, Singapore; 4 Department of Physiology, Yong Loo Lin School of Medicine, National University of Singapore, Singapore; 5 National University Health System (NUHS), Centre for Healthy Longevity, Singapore; 6 Departments of Medicine and Physiology, Yong Loo Lin School of Medicine, National University of Singapore, Singapore

**Keywords:** exercise, obesity, hepatic physiology, skeletal muscle, fat

## Abstract

Investigations of long-term exercise interventions in humans to reverse obesity is expensive and is hampered by poor compliance and confounders. In the present study, we investigated intrahepatic and muscle fat, visceral and subcutaneous fat pads, plasma metabolic profile and skeletal muscle inflammatory markers in response to 12-week aerobic exercise in an obese rodent model. Six-week-old male Wistar rats (*n*=20) were randomized to chow-fed control (Control, *n*=5), sedentary high-fat diet (HFD, *n*=5), chow-fed exercise (Exercise, *n*=5) and HFD-fed exercise (HFD+Exercise, *n*=5) groups. The exercise groups were subjected to 12 weeks of motorized treadmill running at a speed of 18 m/min for 30 min/day. Differences in post-intervention measures were assessed by analysis of covariance (ANCOVA), adjusted for baseline bodyweight and pre-intervention measures, where available. Post-hoc analyses were performed with Bonferroni correction. Plasma metabolic profile was worsened and fat pads, ectopic fat in muscle and liver and inflammatory markers in skeletal muscle were elevated in sedentary HFD-fed animals relative to chow-fed controls. HFD+Exercise animals had significantly lower leptin (*P*=0.0004), triglycerides (*P*=0.007), homeostatic model assessment of insulin resistance (HOMA-IR; *P*=0.065), intramyocellular lipids (IMCLs; *P*=0.003), intrahepatic lipids (IHLs; *P*<0.0001), body fat% (*P*=0.001), subcutaneous adipose tissue (SAT; *P*<0.0001), visceral adipose (*P*<0.0001) and total fat mass (*P*<0.0001), relative to sedentary HFD-fed animals, despite only modestly lower bodyweight. Messenger RNA (mRNA) expression of inflammatory markers Interleukin 6 (IL6) and Tumor necrosis factor α (TNFα) were also reduced with aerobic exercise in skeletal muscle. Our results suggest that 12 weeks of aerobic exercise training is effective in improving metabolic health, fat depots, ectopic fat and inflammation even against a high-fat dietary background.

## Introduction

Obesity and its associated comorbidities represent major challenges to human health in the 21^st^ century [1]. The increasing prevalence of obesity is likely to be a consequence of the combination of the modern sedentary lifestyle with the modern obesogenic diet. While obesity represents a state of fat-excess, the metabolically deleterious effects of obesity may specifically stem from increased fat accumulation in pathogenic depots, specifically in the abdominal compartments [2] and ectopic sites like liver and muscle [3]. The abdomen is a major site for accumulation of fat including subcutaneous and visceral fat depots. It is the primary source for circulating free fatty acids and has been strongly linked to metabolic dysfunction [2,4]. Accumulation of fat in hepatocytes (fatty liver disease) is associated with impaired hepatic glucose and fat metabolism leading to hepatic/systemic insulin resistance [5,6]. Chronic fatty liver disease can also progress to steatohepatitis, fibrosis and cirrhosis. Fatty liver with compromised insulin clearance can lead to hyperinsulinemia [7]. Intramyocellular lipid (IMCL) is formed from the accumulation of lipid droplets within muscle cells and is associated with altered insulin signaling pathways, insulin resistance and risk of type 2 diabetes mellitus [8,9]. IMCL accumulation is also related to dysregulation of fatty acid metabolism due to obesity-related mitochondrial dysfunction [10,11].

Exercise interventions can remodel adipose tissues, including visceral and subcutaneous fat depots [12–14]. Aerobic exercise increases energy expenditure and improves metabolic function. IMCL localized near the mitochondria can be mobilized in response to exercise intervention [15]. Aerobic exercise is also a positive regulator of mitochondrial biogenesis and mitochondrial function in skeletal muscle, whereas the skeletal muscle in sedentary adults is found to have reduced mitochondrial number [16,17]. Chronic exercise can also reduce fat accumulation in the liver [18–21]. Epidemiological evidence suggests that physically active individuals have a 30–50% lower risk of developing type 2 diabetes or cardiovascular disease (CVD), compared with sedentary individuals [22].

When diagnosed and managed proactively, the possibilities of recovery and prevention of costly complications due to obesity are substantial. Exercise is the cornerstone for most anti-obesity interventions. However, running clinical trials for evaluating effects of long-term exercise on metabolic health, body composition and body fat partitioning in humans is hampered by high cost, poor adherence, high attrition, and confounding by genetic, socio-economic and dietary factors [23–27]. Rodent models offer an excellent opportunity to investigate the above question, by ensuring adherence to a specific exercise paradigm and by offering better control of background genetic and dietary confounders. The goal of the present study is to investigate if a 12-week aerobic exercise paradigm can reduce fat pads, fat accumulation at ectopic sites and metabolic parameters in an obese rodent model. Findings from the study can inform exercise paradigms for clinical exercise trials in humans.

## Materials and methods

### Animals diet and exercise intervention

**Figure 1:**
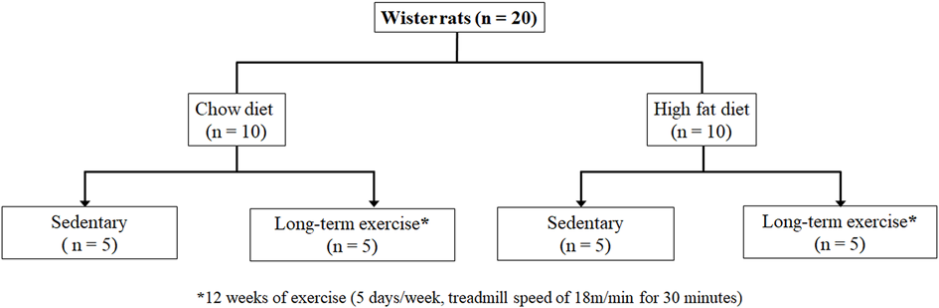
Flow chart of the study plan Flow chart of the experimental design for sedentary and exercise intervened animals fed with chow and HFD.

All experimental procedures and animal research in the present study were approved by A*STAR’s local institutional animal care and use committee (IACUC, approval number# 181350), Singapore. All the animal imaging work was carried out at the Singapore Bioimaging Consortium, and exercise interventions were carried out at the Biological Resource Centre (BRC), Singapore. Animals were acquired from InVivos (Singapore) and housed in a designated holding room controlled for light/dark cycle, temperature, humidity (30–70%) and air quality. The flow chart (Figure 1) depicts the experimental design. All animals used in the study were ordered from same source and were littermates. Six-week-old male Wistar rats (*n*=20) were allocated to one of following four groups using a spreadsheet-generated random number sequence: chow-fed controls (Control, *n*=5), sedentary high-fat diet (HFD, *n*=5), chow-fed exercise (Exercise, *n*=5) and HFD-fed exercise (HFD+Exercise, *n*=5) groups. The HFD and HFD+Exercise groups animals were fed HFD (D12079B, Research Diet, Inc. New Brunswick, NJ) for 12 weeks. The diet compositions are shown in Supplementary Table S1.

Exercise intervention was performed in the chow-fed and HFD-fed exercise groups using a motorized treadmill (model Exer-3/6, Columbus Instruments, OH). Prior to exercise intervention, animals were habituated by allowing them to run on a treadmill for 5 days. During days 1 and 2 of the habituation period, animals were made to run at a speed of 12 and 14 m/min for 30 min, respectively. During days 3–5, treadmill speed was adjusted to 18 m/min for 30 min. After the habituation period, treadmill running was performed by animals in these groups with a speed of 18 m/min for 30 min daily for 12 weeks.

### Physiological measurements

Physiological measurements; bodyweight and blood sampling, were performed at baseline (prior to diet/exercise intervention), and after 12 weeks of diet or exercise intervention. Animals were fasted for 12 h before blood plasma was collected. Leptin and insulin measurements were made using rat Leptin ELISA kit (Uscn Life Science Inc. China, Coefficient of Variation (CV) < 12%) and an ultra-sensitive rat insulin ELISA kit (Crystal Chem, Inc. IL, CV < 10%). Glucose measurements were performed by Quest Laboratories Pte Ltd (Singapore) using an ADVIA Chemistry glucose hexokinase_3 (GLUH_3) reagent kit (Siemens Healthcare Diagnostics Inc. NY, CV < 10%).

### Homeostatic model assessment of insulin resistance determination

Animals were fasted for 12 h and blood samples were collected pre- and post-exercise interventions. The plasma insulin and plasma glucose were evaluated to calculate the Homeostatic Model Assessment of Insulin Resistance (HOMA-IR) [28]. Blood samples for insulin were collected from the rat tail vein using a needle prick and placed directly into an Eppendorf tube. It was then immediately placed in an ice-bath and centrifuged at 2000×***g*** for 15 min at room temperature, according to the manufacturer’s instructions (Insulin - Crystal Chem, Inc. IL; Glucose - Siemens Healthcare Diagnostics Inc. NY) Plasma was stored at −80°C. Rat insulin assay determinations were done by the luminescence method (Luminex™) using kits purchased from ultra-sensitive rat insulin ELISA kit (Crystal Chem, Inc. IL). Blood glucose was determined by Quest Laboratories Pte Ltd (Singapore) using an ADVIA Chemistry GLUH_3 reagent kit (Siemens Healthcare Diagnostics Inc. NY). HOMA-IR was determined by the formula [28]:

$$\text{HOMA IR}=\frac{\text{ glucose concentration (mg/dl) }\times \;\text{ insulin concentration }(\text{μU/ml})}{405}$$

### *In vivo* magnetic resonance spectroscopy

Magnetic resonance spectroscopy (MRS) measurements for assessing ectopic fat in liver and muscle were performed both pre- and post-intervention. MRS measurements were performed using a 7T Bruker ClinScan system with a 72-mm volume transmit and 20-mm receive only coil. Animals were initially anesthetized with 2.5–3% of isoflurane in combination with medical grade oxygen and air in a dedicated chamber prior to imaging, and later reduced to 1.5–2.0% to maintain the respiration within 60–65 cycles/min during the imaging experiments. Respiration and body temperature were monitored using a physiological monitoring system (ML880 16/30 power lab system, AD Instruments, Spechbach, Germany).

Liver spectra was obtained from a 4 × 4 × 4 mm^3^ voxel placed in the right lobe of the liver with volume localized point resolved spectroscopy sequence (PRESS) with and without water suppression (TR = 4000 ms, TE = 13 ms, 64 averages for water-suppressed and 4 averages for water-unsuppressed, 2048 complex points, spectral width = 4500 Hz and respiratory gating with a trigger delay of 35 ms). Water-suppressed and unsuppressed spectra were also obtained from the tibialis anterior muscle from a 3 × 3 × 3 mm^3^ voxel using the PRESS sequence (TR = 4000 ms, TE = 13 ms, 128 averages for water-suppressed and 4 averages for water-unsuppressed, 2048 complex points and spectral width = 4500 Hz).

### Data processing and analysis

The quantification of liver and muscle data was performed using Linear combination model fitting (LCModel) [29] as described in our earlier work [30]. The unsuppressed water signal was utilized for eddy current correction. The intrahepatic lipid (IHL) content was estimated using the liver fat fraction, which was calculated as the ratio of the lipid peak area to the sum of lipid and unsuppressed water peak areas. The water and lipid peak areas were corrected for T_2_ losses before calculating the fat fraction. The skeletal muscle IMCL content was measured as the ratio of the IMCL methylene peak normalized by the Creatine 3.0 ppm peak [30].

### Tissue extraction and biochemical analysis

After terminal MR imaging measurements, animals were euthanized in a closed chamber with increasing concentration of carbon dioxide. Various tissues were surgically collected from the liver, tibialis muscle and fat tissues including visceral adipose tissue (VAT) compartments (gonadal, mesenteric and retroperitoneal, perirenal) and subcutaneous adipose tissue (SAT). Tissues were weighed and frozen in liquid nitrogen and stored at −80°C for messenger RNA (mRNA) analysis. The total fat pad mass and the bodyweight were used to estimate the percentage of body fat.

### mRNA analysis by quantitative polymerase chain reaction

Total RNA was isolated from the tibialis muscle using the RNeasy Kit (Qiagen) according to the manufacturer’s recommendations. RNA quality was checked using the Nanodrop method. Complementary DNA was synthesized using Superscript Vilo reverse transcriptase (Life Technologies), quantitative polymerase chain reaction (qPCR) was performed in triplicates using PCR Master Mix (Invitrogen, Thermo Fisher Scientific). Relative gene expression of inflammatory markers, Interleukin 6 (IL6) and Tumor necrosis factor α (TNFα), were analyzed with the iCycler Thermal Cycler (Bio-Rad) using ∆∆^−*C*_t_^ method with β-actin as the loading control [31–33]. mRNA-fold changes were expressed with respect to the control group. The sequences of the qPCR primers used were IL6 forward-TCCTACCCCAACTTCCAATGCTC and IL6 reverse-TTGG ATGGTCTTGGTCCTTAGCC, TNF-α forward AAATGGGCTCCCTCTCATCAGTTC and TNF-α reverse TCTGCTTGGTGGTTTGCTACGAC and Actin Forward-AAGTC CCTCACCCTCCCAAAAG and reverse-AAGCAATGCTGTCACCTTCCC [34].

### Statistical analysis

Inspection of the baseline data (shown in Supplementary Table S2) indicated significant differences in bodyweight prior to intervention. To take this baseline heterogeneity into account, group differences in post-intervention measurements were evaluated by after adjusting for baseline bodyweight using analysis of covariance (ANCOVA). Bonferroni correction was used for post-hoc pairwise comparisons. Further, for measurements performed both pre- and post-intervention (biochemical measurements and MRS measurements), we additionally included the pre-intervention measurement as a covariate in the ANCOVA models. A threshold of *P*=0.05 was used for assessing statistical significance.

### Blinding

Since the same set of researchers were involved in both running the experimental interventions and performing the data analysis, they were not blinded to the grouping.

## Results

**Figure 2:**
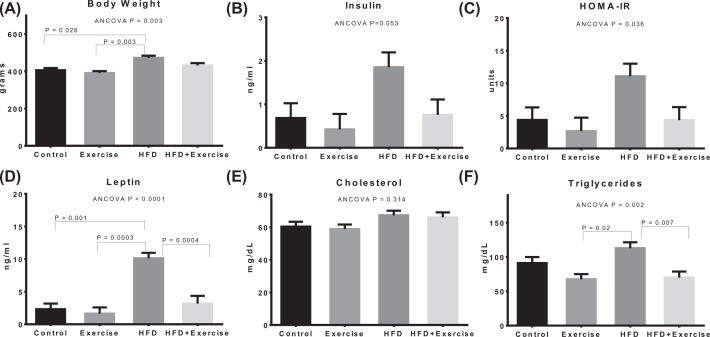
Bodyweight and biochemical measurements Estimated marginal means of (**A**) bodyweight and biochemical measurements including (**B**) insulin, (**C**) HOMA-IR, (**D**) leptin, (**E**) cholesterol and (**F**) triglyceride, after adjustment for baseline covariates (bodyweight and pre-intervention measurement). Error bars indicate standard error of mean. Statistical significance is shown (*P*<0.05) for pairwise associations after Bonferroni correction.

**Figure 3:**
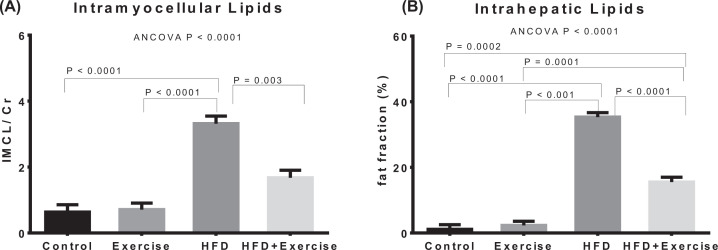
Quantification of lipids from liver and skeletal muscle Estimated marginal means of (**A**) IMCLs, (**B**) IHLs after adjustment for baseline covariates (bodyweight and pre-intervention measurement). Error bars indicate standard error of mean. Statistical significance is shown (*P*<0.05) for pairwise associations after Bonferroni correction.

**Figure 4:**
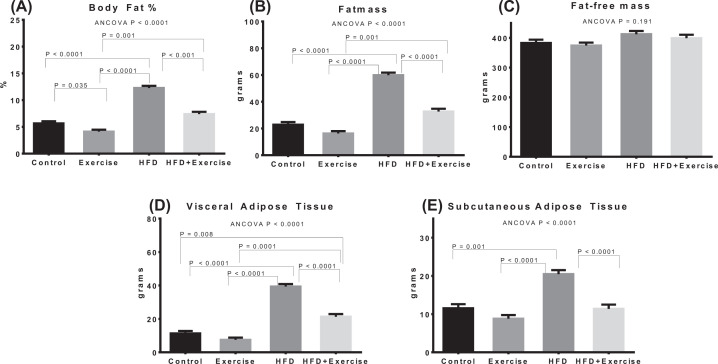
Quantification of abdominal fat depots Estimated marginal means of body fat % (**A**), fatmass (**B**), fat-free mass (**C**), fat pad measurements from VAT (**D**) and SAT (**E**), after adjustment for baseline bodyweight. Error bars indicate standard error of mean. Statistical significance is shown (*P*<0.05) for pairwise associations after Bonferroni correction.

**Figure 5:**
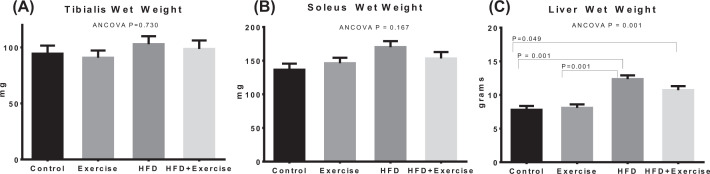
Quantification of skeletal muscle and liver wet weights Estimated marginal means of the wet weights of tibialis muscle (**A**), soleus muscle (**B**) and liver (**C**) after adjustment for baseline bodyweight. Error bars indicate standard error of mean. Statistical significance is shown (*P*<0.05) for pairwise associations after Bonferroni correction.

Estimated marginal means after adjustment for baseline covariate using ANCOVA are shown for bodyweight and biochemical measurements (Figure 2), ectopic fat (IMCL and IHL) assessments (Figure 3), fat pads and adiposity assessments (Figure 4), and organ wet weights (Figure 5) of soleus muscle, tibialis muscle and liver. The ANCOVA analysis revealed statistically significant group differences for bodyweight (*P*=0.003), HOMA-IR (*P*=0.036), leptin (*P*<0.0001) and triglycerides (*P*=0.002), ectopic fat, visceral and subcutaneous fat pads and body fat % (*P*<0.00001) and liver wet weight (*P*=0.001). Relative to the control group, the HFD group had significantly elevated bodyweight (*P*=0.028) and adiposity-related measures, namely, leptin (*P*=0.001), IMCL (*P*<0.0001), IHL (*P*<0.0001), body fat percentage (*P*<0.00001), total fat mass (*P*<0.0001), VAT (*P*<0.0001), SAT (*P*=0.001) and liver wet weight (*P*<0.001). However, elevations in total fat-free mass and tibialis and soleus muscle wet weights were not significant. The HOMA-IR in the HFD groups was more than 2.5-fold higher than the control group but the difference was not statistically significant (*P*=0.282). Within the HFD fed animal, exercise was found to result in significantly lower leptin (*P*=0.0004), triglycerides (*P*=0.007), IMCL (*P*=0.003), IHL (*P*<0.0001), body fat% (*P*=0.001), SAT (*P*<0.0001), VAT (*P*<0.0001) and total fat mass (*P*<0.0001). The above changes occurred in spite of the fact that the body-weight was not significantly lower. HOMA-IR was lower by nearly 60% in the HFD+Exercise group relative to the HFD group, although the difference was borderline significant (*P*=0.065). The mean, standard error of the mean, and individual datapoints for the above post-intervention assessments, without adjustment for baseline covariates, are shown in the Supplementary Figures S3–S6.

**Figure 6:**
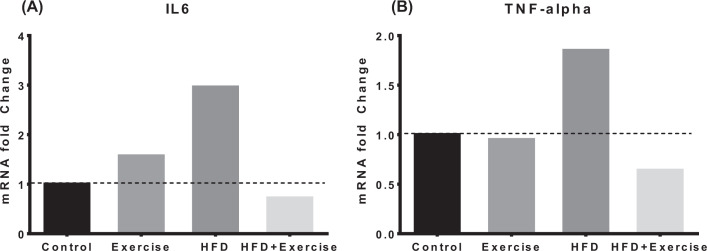
mRNA expression of inflammatory markers from skeletal muscle mRNA expression of inflammatory makers IL-6 (**A**) and TNF-α (**B**) from tibialis muscle relative to controls using ∆∆^−*C*_t_^ method.

The mRNA fold-changes for the inflammatory markers are shown in Figure 6. Relative to the control group, HFD group had markedly elevated TNF-α (fold-change = 1.85) and IL6 (fold-change = 2.96). However, the HFD+Exercise group had lower levels of TNF-α (fold-change = 0.64) and IL6 (fold-change = 0.72) compared with the control group.

Representative spectra from muscle and liver, pre- and post-exercise are shown in the Supplementary Figures S1 and S2, respectively.

## Discussion

There is considerable clinical interest in implementing exercise and dietary interventions to combat obesity and diabetes [35–37]. However, implementing long-term exercise studies in humans is quite challenging due to high cost, poor compliance, high attrition and several confounding factors [23–27]. In the present study, we evaluated the metabolic effects of 12 week of aerobic exercise for improving blood lipid and glucose profiles, skeletal muscle and liver lipid metabolism and inflammatory markers in a rodent model of obesity. The bodyweight, adiposity-related measures (fat pads, ectopic fat in liver and muscle, body fat % and leptin), muscle inflammatory markers and HOMA-IR were elevated in sedentary HFD fed animals relative to controls. However, HFD-fed animals which underwent a 12-week aerobic exercise intervention had an improved plasma metabolic profile (leptin, triglycerides and HOMA-IR) and lower ectopic fat accumulation in liver and muscle, fat pads as well as inflammatory markers in the muscle, relative to the sedentary HFD-fed animals.

There could be several metabolic adaptations to long-term exercise that could explain our findings in the present study. During aerobic exercise, the activity of lipoprotein lipase (LPL)–lipoprotein increases and hydrolyzes circulating triglycerides, chylomicrons and lipoproteins. Hence, it is likely that the increased activity of LPL [38] and utilization of blood lipids resulted in significant reduction in plasma lipids in exercised animals. At rest, the fuel source is predominantly free fatty acids which switches during aerobic exercise to either fat, glucose or amino acids, depending on exercise intensity [39,40].

IHL, which was markedly elevated in the sedentary HFD animals, is known to be a major risk factor for type 2 diabetes. Liver is the dominant ectopic organ for fat disposal in a sedentary state with high calorie intake. Excessive accumulation of triglycerides in hepatocytes is associated with increased fatty acid delivery from VAT, increased *de novo* lipogenesis and impaired triglyceride export [41,42]. Excessive accumulation of IHL in HFD group could be due to increased uptake of circulating fatty acids derived from the elevated VAT under sedentary conditions. Exercise adaptation involves the reduction in both subcutaneous and VAT mass and/or enhanced adipose insulin sensitivity resulting in lower uptake of free fatty acids and also facilitates the fatty acid disposal through oxidation/ketogenesis [20]. The lower IHL in the HFD+Exercise group may indicate well-controlled uptake of free fatty acids with reduced lipogenesis in the liver [19]. Interestingly the reduction in IHL occurred even though the reductions in bodyweight relative to HFD group were quite modest. This is concordant with studies indicating that exercise interventions can reduce IHL without changing bodyweight body [18,21,43].

Skeletal muscle takes up the circulating plasma free fatty acids by up-regulation of: fatty acid translocase, plasma membrane fatty acid binding protein (FABP4) and by increased lipolytic action of LPL [44]. Under sedentary conditions, most of the free fatty acids are stored (90%) in the soleus muscle as IMCL [45,46]. IMCL was markedly elevated in the sedentary HFD group. Increased IMCL in the HFD group could be due to increased availability of circulating free fatty acids [47] and impaired lipid oxidation [48]. The addition of aerobic exercise to HFD resulted in a significant reduction in IMCL relative to the sedentary HFD group. During the aerobic exercise, skeletal muscle IMCL and circulating lipoproteins are the main fuel sources [49,50]. During the exercise, there is increased whole-body oxygen consumption (VO_2_), which is facilitated by enhanced blood flow from the increased cardiac output. This enables the effective delivery of oxygen and lipids as fuel substrates for the skeletal muscle for enhanced mitochondrial respiration to fulfil energy demands. During 12-week aerobic exercise, the skeletal muscle undergoes adaptive response for handling the increased capacity of fatty acid substrates at the expense of glucose, and thereby leads to enhanced fatty acid oxidation [51]. Twelve-week aerobic exercise training enhances oxidative metabolism of skeletal muscle by increasing the abundance of mitochondrial enzymes [52,53]. During exercise, skeletal muscle behaves as an endocrine organ, secreting various myokines, such as myostatin and its concentration decreases in the blood and muscle [54]. A decrease in exercise-induced myostatin is associated with reduction in muscle fat and improves the glucose metabolism [55]. Aerobic exercise also increases the protein expression of glucose transporter proteins and increased mitochondrial biogenesis thereby improving mitochondrial function.

Increased adipose tissue mass and ectopic fat are also strongly linked to insulin resistance, inflammation and risk of type 2 diabetes [2–4]. We found that aerobic exercise in HFD-fed animals resulted in lower adipose tissue as well as ectopic fat relative to sedentary HFD-fed animals. This could potentially explain the lower insulin resistance and inflammatory markers we observed in the HFD-fed animals undergoing aerobic exercise.

## Conclusion

Our results show that 12 weeks of aerobic exercise resulted in lower plasma leptin, triglycerides and HOMA-IR in HFD-fed rats relative to sedentary HFD-fed rats. The intervention also resulted in lower IHL, abdominal fat depots and skeletal muscle IMCL. The inflammatory markers IL6 and TNFα, which were markedly higher in HFD-fed sedentary rats, were also lower with exercise intervention. These findings have implications for clinical translation and suggest that incorporating long-term aerobic exercise may be beneficial for improving metabolic health in the context of a modern obesogenic environment.

## Supplementary Material

Supplementary Figure S1-S6 and Tables S1-S2

## Data Availability

The authors confirm that the data supporting the findings are available within the article and its supplementary materials.

## Abbreviations

ANCOVA: analysis of covariance
CV: coefficient of variation
GLUH_3: glucose hexokinase_3
HFD: high-fat diet
HOMA-IR: homeostatic model assessment of insulin resistance
IHL: intrahepatic lipid
IL6: interleukin 6
IMCL: intramyocellular lipid
LPL: lipoprotein lipase
mRNA: messenger RNA
MRS: magnetic resonance spectroscopy
PRESS: point resolved spectroscopy
qPCR: quantitative polymerase chain reaction
SAT: subcutaneous adipose tissue
TE: echo time
TNFα: tumor necrosis factor α
TR: time of repetition
VAT: visceral adipose tissue
