# Supplementary material for: A 12-week aerobic exercise intervention results in improved metabolic function and lower adipose tissue and ectopic fat in high-fat diet fed rats
Source: Biosci Rep. 2021 Jan 29;41(1):BSR20201707. doi: 10.1042/BSR20201707 (PMC7846962; doi:10.1042/BSR20201707)
Supplement: Supplementary Figure S1-S6 and Tables S1-S2 [file BSR-2020-1707_supp.pdf]

Supplementary Section

Supplementary Table. 1. Diet composition

Composition of control diet (10% kilocalories including 17% protein, 73% carbohydrate, and 10% fat) and high fat diet (40% kilocalories including 17% protein, 43% carbohydrate, and 40% fat) in gm. (Research Diet Inc)

**Control diet**		**High fat diet (HFD) (D12079B)**	
Oil	6.0	Corn oil	0.9985
Casein	24.5	Casein- Vitamin free	19.4700
Sucrose	10.0	Sucrose	34.0476
Starch	46.5	Corn starch	4.9923
Vitamin mix (AIN 76)	1.0	Vitamin mix (AIN76)	0.9985
Mineral mix (Oriental Yeast)	7.0	Mineral mix (AIN76)	3.4946
Cellulose powder	5.0	Cellulose powder	4.9923
		Maltodextrin	9.9846
		Calcium carbonate	0.3994
		DL-Methionine	0.2995
		Choline Bitartrate	0.1997
		Cholesterol	0.1498
		Ethoxyquin	0.0040
		Milk fat	19.9692

Supplementary Table. 2. Baseline measurement of blood chemistry and ectopic fat

Baseline physiological measurements and ectopic fat quantification. Group differences at baseline were analyzed by one-way ANOVA.

**Measurements**	**Control**	**Exercise**	**HFD**	**HFD + Exercise**	**P-value**
Body-weight (g)	239.0 ± 9.4	247.2 ± 9.1	283.8 ± 5.6	283.0 ± 7.4	0.002
Insulin (ng/ml)	0.44 ± 0.05	0.79 ± 0.18	0.35 ± 0.05	0.50 ± 0.08	0.042
HOMA-IR (units)	2.44 ± 0.29	4.80 ± 1.03	2.00 ± 0.32	2.79 ± 0.48	0.024
Leptin (ng/ml)	1.67 ± 0.02	1.59 ± 0.02	1.72 ± 0.02	1.85 ± 0.05	1.9E-4
Cholesterol (mg/dL)	61.20 ± 5.89	73.80 ± 6.19	55.80 ± 1.20	52.20 ± 4.61	0.033
Triglycerides (mg/dL)	73.80 ± 8.19	75.00 ± 7.16	61.80 ± 3.75	61.80 ± 7.74	0.376
IHL (%)	1.63 ± 0.38	0.92 ± 0.08	1.73 ± 0.32	1.12 ± 0.11	0.366
IMCL/Cr	0.93 ± 0.07	1.00 ± 0.12	1.91 ± 0.90	0.89 ± 0.05	0.133

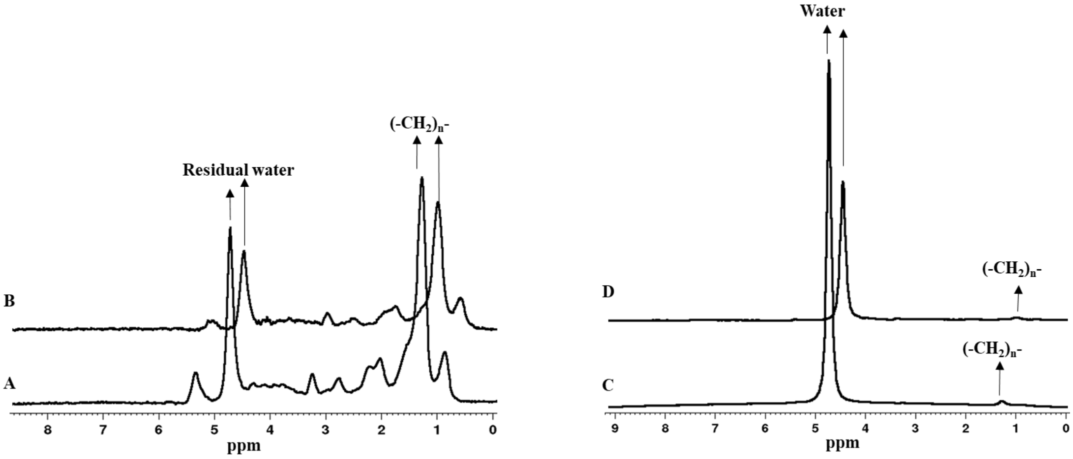

Supplementary Figure 1. Representative liver spectra with and without exercise intervention.

Liver spectra is shown before and after exercise intervention from a rat fed with chow diet. Baseline liver spectrum is shown in Figure 1A and Figure 1B shows the spectrum with exercise intervention. The unsuppressed water signal is shown without (Figure 1C) and with (Figure 1D) exercise intervention.

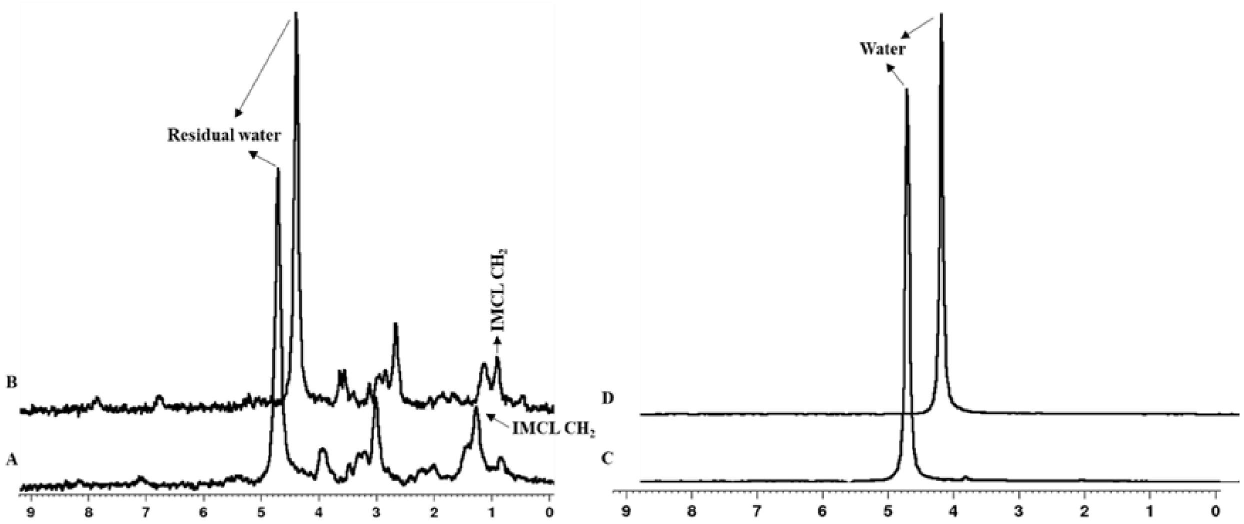

Supplementary Figure 2. Representative muscle spectra with and without exercise intervention.

Muscle spectra (Tibialis Anterior) is shown before and after exercise intervention from a rat fed with chow diet. Baseline muscle spectrum is shown in Figure 2A and Figure 2B shows the spectrum with exercise intervention. The unsuppressed water signal is shown without (Figure 2C) and with (Figure 2D) exercise intervention.

,

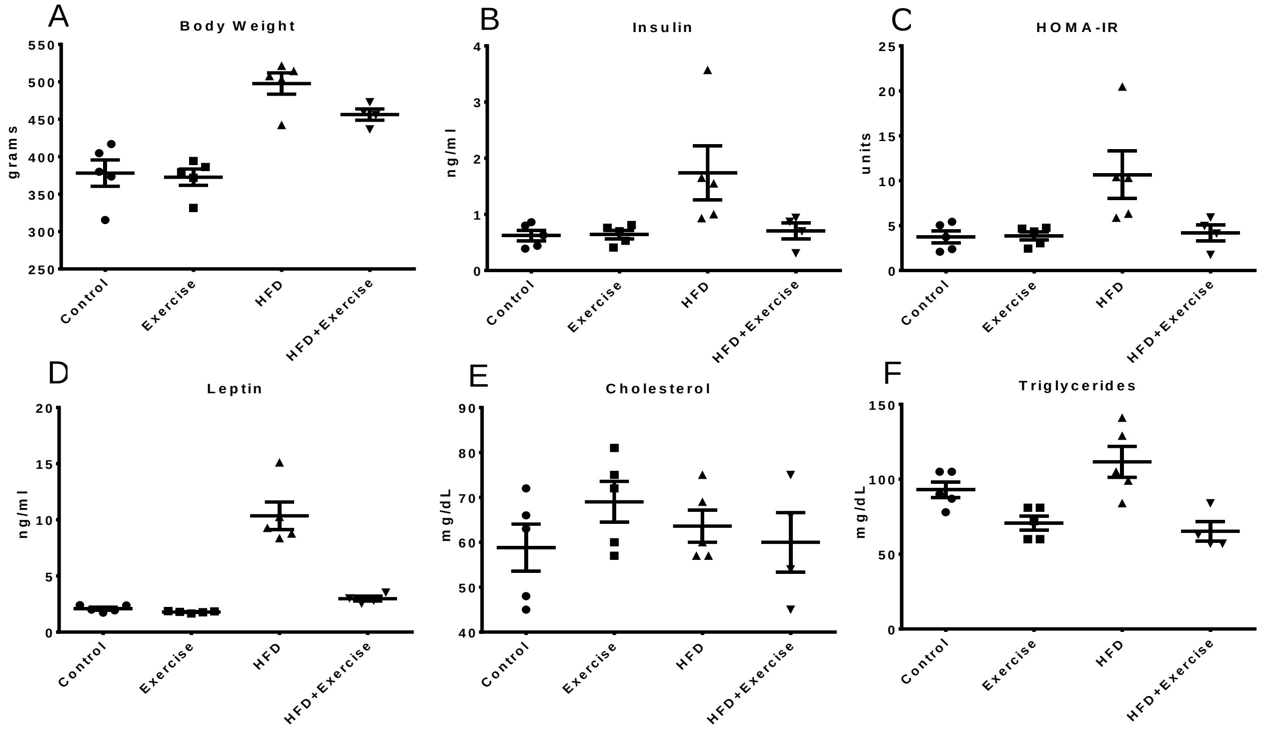

Supplementary Figure 3. Body weight and biochemical measurements before and after exercise intervention.

Group means of post-intervention (A) body weight and biochemical measurements including (B) insulin, (C) HOMA-IR, (D) leptin, (E) cholesterol and (F) triglyceride. Error bars indicate standard error of mean.

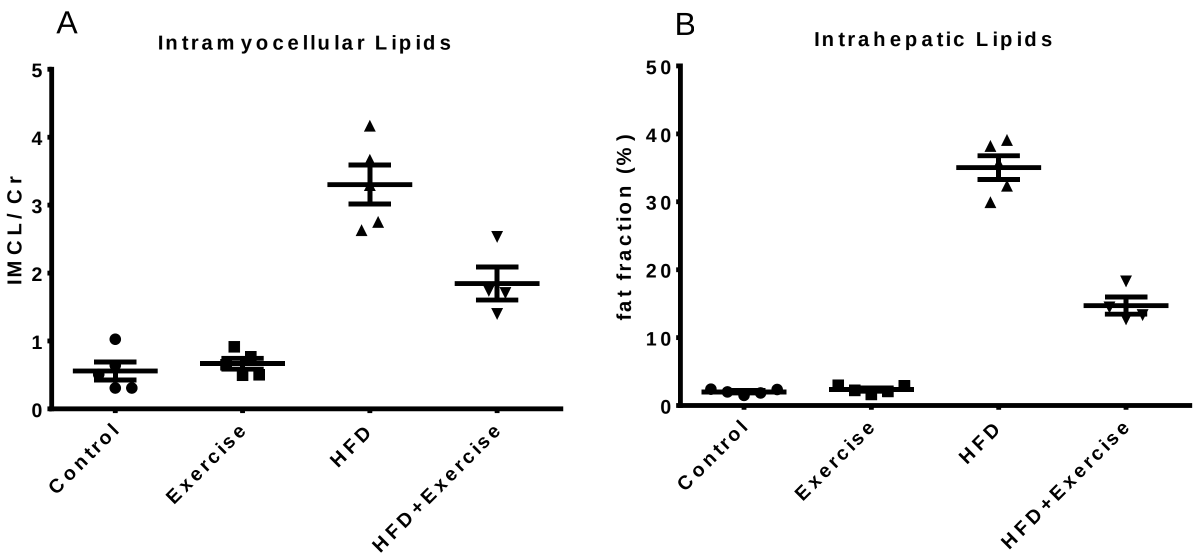

Supplementary Figure 4. Intramyocellular and intrahepatic lipids before and after exercise intervention.

Group means of post-intervention (A) intramyocellular lipids (IMCL) and (B) intrahepatic lipids (IHL). Error bars indicate standard error of mean.

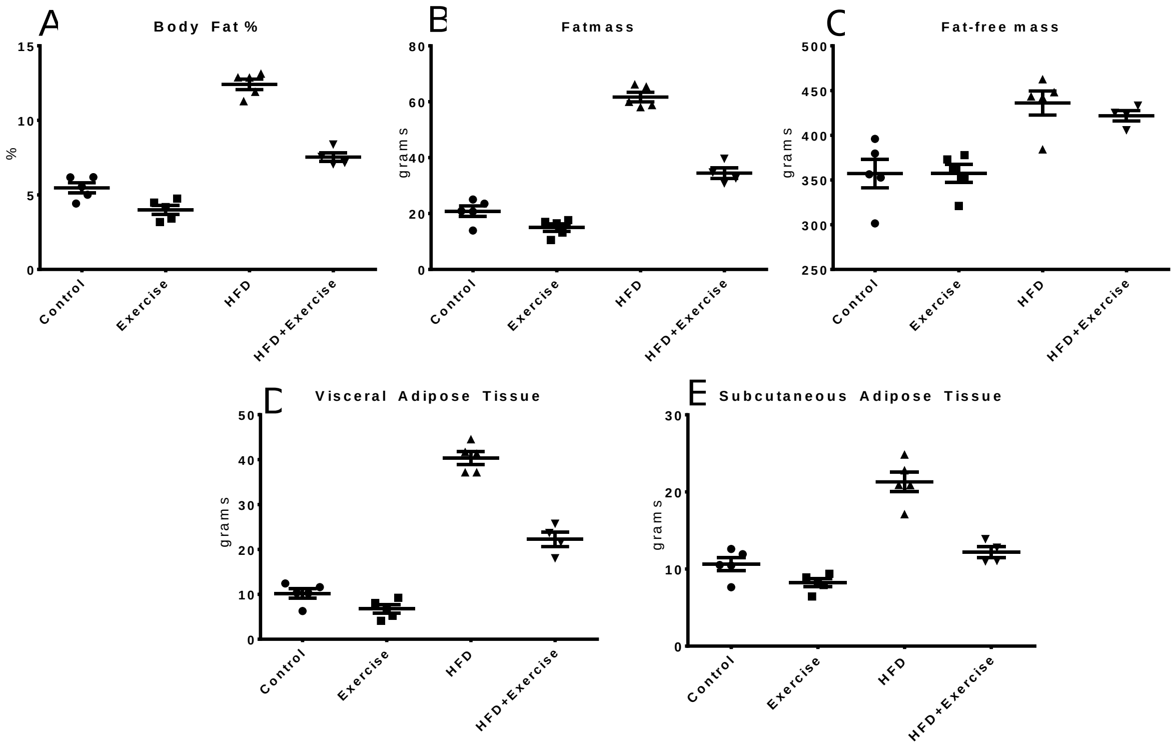

Supplementary Figure 5. Body fat, fat mass and adipose tissue changes before and after exercise intervention.

Group means of post-intervention (A) body fat %, (B) fatmass, (C) fat-free mass fat-pad measurements from (D) visceral adipose tissue and (E) subcutaneous adipose tissue. Error bars indicate standard error of mean.

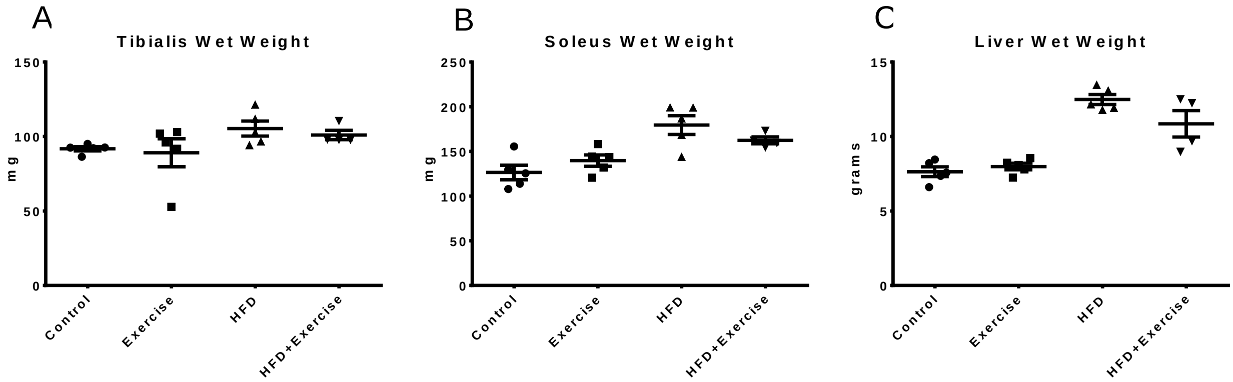

Supplementary Figure 6. Muscle and liver tissue quantification before and after exercise intervention.

Group means of post-intervention wet weights of (A), tibialis muscle (B) soleus muscle and (C) liver. Error bars indicate standard error of mean.
